# Preliminary monitoring of concentration of particulate matter (PM_2.5_) in seven townships of Yangon City, Myanmar

**DOI:** 10.1186/s12199-018-0741-0

**Published:** 2018-10-25

**Authors:** Ei Ei Pan Nu Yi, Nay Chi Nway, Win Yu Aung, Zarli Thant, Thet Hnin Wai, Kyu Kyu Hlaing, Cherry Maung, Mayuko Yagishita, Yang Ishigaki, Tin-Tin Win-Shwe, Daisuke Nakajima, Ohn Mar

**Affiliations:** 1 Department of Physiology, University of Medicine 1, Yangon, Myanmar; 2 Center for Health and Environmental Risk Research, National Institute for Environmental Studies, 16-2 Onogawa, Tsukuba, Ibaraki 305-8506 Japan; 3 Graduate School of Informatics and Engineering, University of Electro-communications, Tokyo, Japan

**Keywords:** PM_2.5_, Pocket Sensor, Regional distribution, Yangon

## Abstract

**Background:**

Airborne particulate pollution is more critical in the developing world than in the developed countries in which industrialization and urbanization are rapidly increased. Yangon, a second capital of Myanmar, is a highly congested and densely populated city. Yet, there is limited study which assesses particulate matter (PM_2.5_) in Yangon currently. Few previous local studies were performed to assess particulate air pollution but most results were concerned PM_10_ alone using fixed monitoring. Therefore, the present study aimed to assess distribution of PM_2.5_ in different townships of Yangon, Myanmar. This is the first study to quantify the regional distribution of PM_2.5_ in Yangon City.

**Methods:**

The concentration of PM_2.5_ was measured using Pocket PM_2.5_ Sensor (Yaguchi Electric Co., Ltd., Miyagi, Japan) three times (7:00 h, 13:00 h, 19:00 h) for 15 min per day for 5 days from January 25^th^ to 29^th^ in seven townships. Detailed information of eight tracks for PM_2.5_ pollution status in different areas with different conditions within Kamayut Township were also collected.

**Results:**

The results showed that in all townships, the highest PM_2.5_ concentrations in the morning followed by the evening and the lowest concentrations in the afternoon were observed. Among the seven townships, Hlaingtharyar Township had the highest concentrations (164 **±** 52 μg/m^3^) in the morning and (100 **±** 35 μg/m^3^) in the evening. Data from eight tracks in Kamayut Township also indicated that PM_2.5_ concentrations varied between different areas and conditions of the same township at the same time.

**Conclusion:**

Myanmar is one of the few countries that still have to establish national air quality standards. The results obtained from this study are useful for the better understanding of the nature of air pollution linked to PM_2.5_. Moreover, the sensor which was used in this study can provide real-time exposure, and this could give more accurate exposure data of the population especially those subpopulations that are highly exposed than fixed station monitoring.

## Background

Particulate matter (PM) is one of the most common air pollutants and a complex mixture of extremely small particles and liquid droplets made up of acids, organic chemicals, metals, and soil or dust particles [1]. Using aerodynamic diameter, PM is divided into PM_10_ (diameter of 10 μm and less), PM_2.5_ (diameter of 2.5 μm and less), and ultrafine (diameter less than 0.1 μm). Sources of PM pollution can be both anthropogenic and natural. Man-made sources include combustion of engines (both diesel and petrol), solid-fuel (coal, lignite, heavy oil, and biomass), combustion for energy production in households and industries, industrial activities (building, mining, manufacture of cement, ceramic, and bricks and smelting), erosion of pavement by road traffic, and abrasion of brakes and tyres [2]. Soil and resuspended dust are also contributing sources of PM particularly in arid areas.

PM_2.5_ are the greatest risks to health, as they are capable of penetrating peoples’ lungs and entering their blood stream [3]. It has been reported that a long-term exposure to PM_2.5_ is associated with an increase in cardiopulmonary mortality by 6–13% per 10 μg/m^3^ of PM_2.5_ [4, 5]. In addition to well-documented effects on respiratory and cardiovascular health, there is evidence linking long-term exposure to PM_2.5_ with adverse birth outcomes [6, 7], diabetes [8], and neurodevelopment and cognitive function [9].

Airborne particulate pollution is more critical in the developing world than in the developed countries. Yangon is formerly known as Rangoon and located in Myanmar which is one of Southeast Asian countries. Yangon is the second capital of Myanmar and major city of Myanmar’s economic areas and more than five millions people occupied there. Yangon City is complex with residential, commercial, and industrial buildings [10]. Recently, due to rapid economic development, increasing motor vehicles, expansion of industries, and urbanization, it is time to assess the air quality in Yangon. Yet, there is limited study which assesses PM_2.5_ in Yangon currently. To make necessary improvement in air quality management in our country that is still in early stage, it is essential to do research on particulate air pollution which is now recognized as a risk factor for public health [11]. Therefore, the present study aimed to assess distribution of PM_2.5_ in different townships of Yangon, Myanmar.

Recently, Ishihaki et al. (2017) [12] devised Pocket PM_2.5_ Sensor, a prototype system of a sensor connected to smart phone, which detects both PM_10_ and PM_2.5_, and it can be used in fixed monitoring as well as mobile sensing. Field experiments for these moveable sensors have been carried out in East Asian countries (Japan, China, and Korea) and showed reliable and accurate data [12]. By using this Pocket PM_2.5_ Sensor, we conducted the preliminary survey to assess the air quality with regard to PM_2.5_ in some Townships of Yangon.

This study is a collaborative work between Department of Physiology, University of Medicine 1, Yangon, Myanmar and National Institute for Environmental Studies (NIES), Tsukuba, Japan. The Pocket PM_2.5_ Sensors are purchased from Yaguchi Electric Co., Ltd., Miyagi, Japan.

## Methods

### Materials

**Fig. 1:**
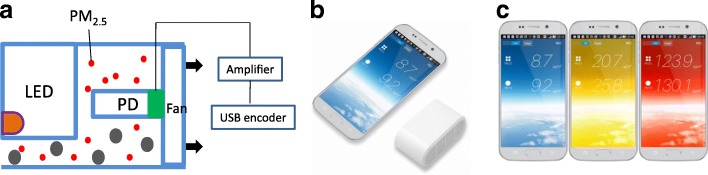
Pocket PM_2.5_ Sensor. **a** Principle of Pocket PM_2.5_ Sensor Module, LED, light-emitting diode; PD photodiode; USB, Universal Serial Bus. **b** Pocket PM_2.5_ Sensor connected to a smart phone with android system. **c** Color variations for different levels of PM_2.5_ concentration. (Adapted from Ishigaki et al. 2017, with permission from Yang Ishigaki)

Pocket PM_2.5_ Sensors (Yaguchi Electric Co., Ltd., Miyagi, Japan) are utilized for measurement of PM_2.5_ concentrations PM_2.5_. Principle of Pocket Sensor Module was shown in Fig. 1a. The sensor has a laser LED (light-emitting diode), a PD (photodiode) sensor, a fan, amplifier, and USB (Universal Serial Bus) encoder. The sensor can generate log data in CSV (comma-separated values) of Google KML (Keyhole Markup Language) format including GPS (Global Positioning System). The portable sensor has to be connected to a smart phone with android system (Fig. 1b). The phone displays PM_2.5_ concentrations in microgram per cubic meter and phone screen color changes from blue, yellow, red, purple to black with increasing values of PM (Fig. 1c). The validity or specification of Pocket PM_2.5_ Sensor provided by Yaguchi Electric Corp. is expressed in Table 1. All Pocket PM_2.5_ Sensors were also calibrated with constantly observed PM_2.5_ counter (PM-712, Kimoto Electric Co., Ltd.) of Air Quality Research Station, National Institute for Environmental Studies (NIES), Tsukuba, Japan.

**Table 1:** Specification of Pocket PM_2.5_ Sensor (Yaguchi Electric Corp.)

No	Item	Parameter
1	Measurement parameters	PM2.5,PM10
2	Measurement range	0.0–999.9 μg /m3
3	Rated voltage	5 V
4	Rated current	60 mA ± 10 mA
5	Sleep current	< 4 mA laser and fan sleep
6	Temperature range	Storage environment: − 10 ~ + 50 °C Work environment:− 20 ~ + 60 °C
7	Humidity range	Storage environment: max 90% Work environment: max 70%
8	Air pressure	86 KPa ~ 110 KPa
9	Corresponding time	1 s
10	Serial data output frequency	1 Hz
11	Minimum resolution of particle	< 0.3 μm
12	Counting yield	70%@0.3 μm 98%@0.5 μm
13	Relative error	Maximum of ± 15% and ± 10 μg/m3 25 °C, 50%RH
14	Product size	42.5 × 32 × 24.5 (mm)

### Power requirement

Voltage:4.7 ~ 5.3 V

Power supply: > 1 W

Supply voltage ripple: < 20 mV

### Air sample collection

The present study was carried out in 2 phases, phase I and phase II. Phase I was carried out for five consecutive days (from 25 January 2018 to 29 January 2018). In the phase I, we measured the concentrations of PM_2.5_ in seven townships (Hlaing, Hlaingthayar, Kamayut, Kyimyindine, Pazundaung, South Okkalapa, and Tamwe) of Yangon City. Some areas in these townships were selected randomly. The investigators walked along the road sides of these areas of the city for three times a day (from 7:00 to 7:15 h, from 13:00 to 13:15 h, and from 19:00 to 19:15 h). They collected PM_2.5_ concentrations using Pocket PM_2.5_ Sensor as mobile sensing. They also checked the color of android screen display and noted down locations and sources of emission when color indicated high PM levels. Then, PM_2.5_ concentrations of morning, afternoon, and evening air samples were calculated.

**Fig. 2:**
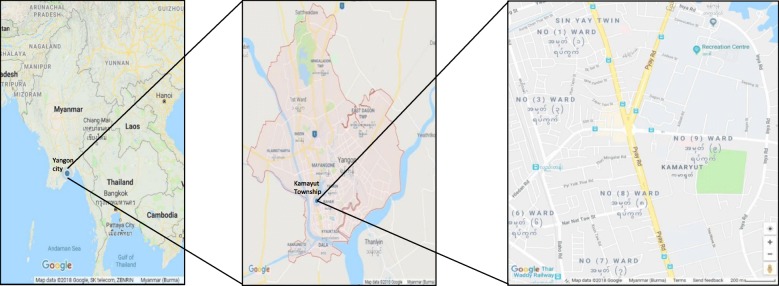
Location of sampling area in Kamayut Township, Yangon, Myanmar. Kamayut Township was selected for the phase II measurement of PM_2.5_ concentration as traffic in this township was congested. (Source from Google map)

Based on the PM_2.5_ concentration among these seven townships, we chose Kamayut Township for the phase II as traffic in this township was congested (Fig. 2). Firstly, we chose the starting point near the Kamayut-Hledan junction and planned eight tracks. Then, eight investigators arrived at the starting point at 15:00 h and recorded the PM_2.5_ concentrations and screen color. Afterwards, they walked along the eight tracts simultaneously for 30 min and collected PM_2.5_ concentrations. Then, we also noted the conditions such as burning leaves or operating generator when PM_2.5_ peak was remarkably high (more than 60 μg/m^3^).

### Validity assessment of Pocket PM_2.5_ Sensor

**Fig. 3:**
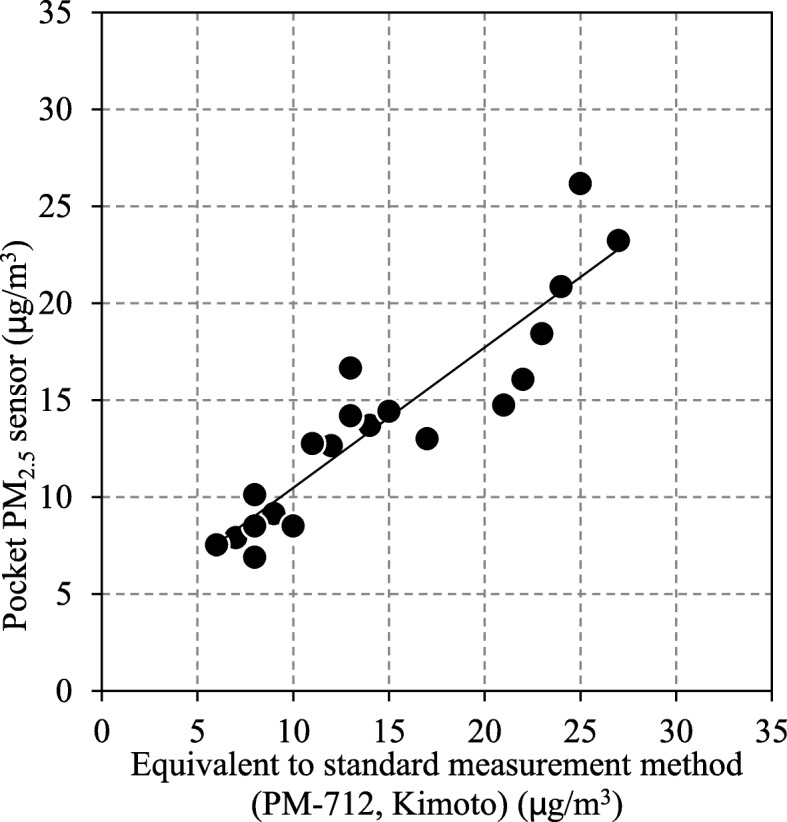
Validity assessment of Pocket PM_2.5_ Sensor: correlation between PM-712, Kimoto and Pocket PM_2.5_ Sensor. To prove validity of Pocket PM_2.5_ Sensor for measuring PM_2.5_ concentration, we compared the data from PM-712, Kimoto, a fixed real-time monitor which was set up in Air Quality Research Station, NIES, Tsukuba, Japan (http://www.nies.go.jp/aqrs/index.html) and Pocket PM_2.5_ Sensor simultaneously. PM-712, Kimoto recorded one hourly while Pocket PM_2.5_ Sensor recorded every second, and we calculated average hour value

To prove validity of our Pocket PM_2.5_ Sensor for measuring PM_2.5_ concentration, we compared the data from PM-712, Kimoto, a fixed real-time monitor which was set up in Air Quality Research Station, NIES, Tsukuba, Japan (http://www.nies.go.jp/aqrs/index.html) and Pocket PM_2.5_ Sensor simultaneously. PM-712, Kimoto recorded one hourly while Pocket PM_2.5_ Sensor recorded every second, and we calculated average hour value. We have shown that a strong correlation exists between PM-712, Kimoto and Pocket PM_2.5_ Sensor in Fig. 3.

### Statistical analysis

Data analysis was done by using Statistical Package for Social Science (SPSS) software version 22. Results were expressed as mean ± SD. For comparison of morning, afternoon and evening PM_2.5_ concentrations in each township, one-way analysis of variance (ANOVA) and post hoc Bonferroni test were used. Statistical significant level was set up at *p* < 0.05.

## Results and discussion

The minimum, maximum, and mean PM_2.5_ concentrations, population density (people/km^2^) and traffic congestion (vehicle/h) of seven townships are shown in Table 2. The concentrations vary during different times of the day, with the maximum concentration of PM_2.5_ in the morning and the minimum concentration in the afternoon in all townships. The highest values in the morning are considered to be due to the smog since study period is a winter season in Yangon, and smoke which is mostly from burning dried leaves and from tea/food shop using log/charcoal burning stove. Zang et al. (2013) [13] have shown that smog is generally caused by high concentrations of fine particles (PM_2.5_) or aerosol.

**Table 2:** PM_2.5_ concentrations (μg/m^3^) of seven townships

Township	Morning (7:00 h) Mean ± SD (Mini~Maxi)	Afternoon (13:00 h) Mean ± SD (Mini~Maxi)	Evening (19:00 h) Mean ± SD (Mini~Maxi)	Population Density (people/km^2^)	Traffic congestion (vehicle/h)	Remark
Hlaing	117 ± 38^Δ^ (66 ~ 458)	38 ± 11* (19 ~ 75)	94 ± 35^♦^ (46~ 246)	9100	Data not available	Residential area
Hlaingtharyar	164 ± 52^Δ^ (94 ~ 287)	31 ± 15* (5 ~ 60)	100 ± 35^♦^ (45~ 186)	10,385	No traffic- light	Industrial area
Kyimyindine	104 ± 69^Δ^ (41 ~ 698)	31 ± 12* (11 ~ 80)	71 ± 27^♦^ (39~ 315)	8955	3600	Semi-residential area
Kamayut	91 ± 37^Δ^ (39 ~ 197)	30 ± 14* (7 ~ 185)	60 ± 22^♦^ (35 ~ 312)	12,000	1920	Semi-residential area
Pazundaung	78 ± 29^Δ^ (31 ~ 192)	35 ± 23* (11 ~ 416)	67 ± 30^♦^ (28 ~ 207)	31,000	3720	Residential area
South Okkalapa	121 ± 35^Δ^ (47 ~ 234)	66 ± 48* (20 ~ 234)	77 ± 29^♦^ (45 ~ 500)	19,635	5760	Commercial area
Tamwe	130 ± 102^Δ^ (49 ~ 878)	39 ± 21* (15 ~ 141)	69 ± 27^♦^ (32 ~ 346)	3200	2520	Residential area

Data are presented as mean ± SD, (minimum~maximum), ANOVA with post hoc: ^Δ^morning vs afternoon, ^♦^morning vs evening, *afternoon vs evening, significant level (*p* < 0.001). Population density data were obtained from Yangon City Development Committee (http://www.ycdc.gov.mm/). Traffic congestion data were recorded at 19:00 h at respective traffic lights in five townships

Motor vehicle emission is one of the major sources of air pollution in urban areas. Bus traffic congestion in the evening (19:00 h) was higher compared to the morning (7:00 h) in Yangon City. Therefore, the evening rise may be due to smoke from vehicle exhaust from higher traffic flows. In addition, both the morning and evening concentrations of PM_2.5_ were found to be higher. Wang et al. (2015) [14] also found that morning and evening peaks in urban areas which are contributed by enhanced anthropogenic activity during rush hour, and the afternoon dip is mainly due to a higher atmospheric mixing layer, which is beneficial for air pollution diffusion. The lowest concentrations in the afternoon is consistent with Harrison et al. 2012 [15] in which the most plausible explanation for the afternoon dip is loss of semi-volatile PM (principally nitrate, with some organic compounds) from the ambient PM, as a result of the higher temperature during this part of the day.

**Fig. 4:**
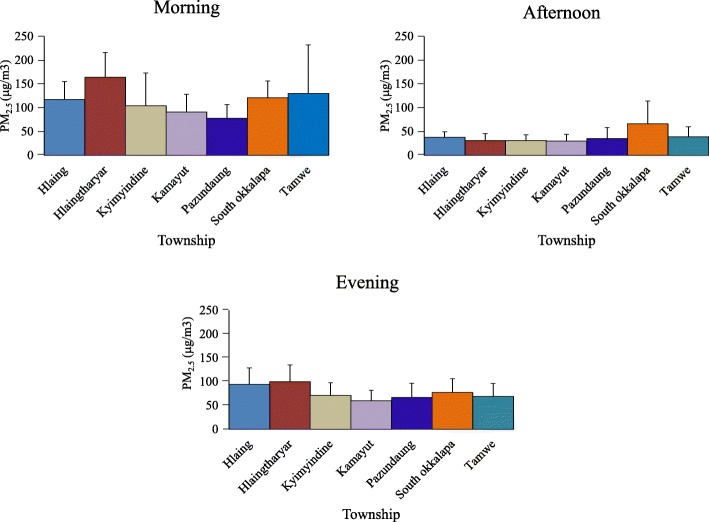
Distribution of PM_2.5_ concentration in seven townships, Yangon, Myanmar. The concentrations of PM_2.5_ in seven townships (Hlaing, Hlaingthayar, Kamayut, Kyimyindine, Pazundaung, South Okkalapa, and Tamwe) of Yangon City were measured. The investigators walked along the road sides of these areas of the city for three times a day (from 7:00 to 7:15 h, from 13:00 to 13:15 h and from 19:00 to 19:15 h). Each bar represents the mean ± SD

Among seven townships, Hlaingtharyar had the highest PM_2.5_ concentrations in the morning (164 **±** 52 μg/m^3^) and the evening (100 **±** 35 μg/m^3^) (Fig. 4). In fact, the selected area in Hlaingthayar Township is in the quiet residential area, but this residential area is located about 300 m away from Yangon-Pathein Highway road and is also surrounded by industrial zone with many factories (i.e., household product factories, food and beverages product factories, and agricultural product factories). So, it indicates that emission from factories and vehicles from highway roads have high impact on particle pollution in the nearby residential area. The highest afternoon PM_2.5_ concentration was in South Okkalapa Township (66 **±** 48 μg/m^3^) may be due to the fact that the selection path was the main road with heavy traffic flow.

**Fig. 5:**
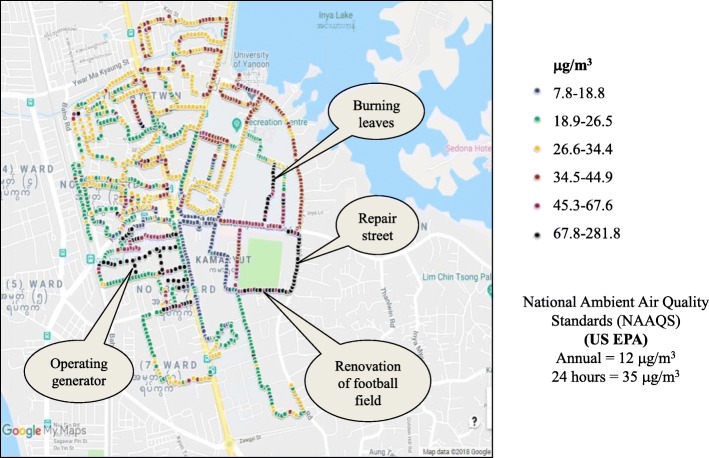
Mapping of PM_2.5_ in Kamayut Township, Yangon, Myanmar. The starting point was near the Kamayut-Hledan junction and planned eight tracks. Eight investigators arrived at the starting point at 15:00 h and recorded the PM_2.5_ concentrations and screen color. Afterwards, they walked along the eight tracks simultaneously for 30 min and collected PM_2.5_ concentrations. (Plotted on Google map)

Regarding eight tracks within Kamayut Township, it was found that PM_2.5_ concentrations varied along with the immediate environment where the investigators walked. Its concentration was found to be low although the investigator was walking in the sun along the road with thick traffic flow. However, when the investigator walked in the residential area and the area with many trees, PM_2.5_ concentration was observed to be high. The highest range of 67.8–281.8 μg/m^3^ was recorded in the places where road repairs were being made and areas where dried leaves were being burned, generator operating, and football field renovated (Fig. 5). In phase I, we also found that PM_2.5_ level was the lowest in the afternoon and relatively higher in the morning and evening. Seasonal and diurnal variations of PM_2.5_ concentration were found in Beijing, Shanghai, and Guangzhou cities in China [16]. In the present study, we found prominent diurnal variation of PM_2.5_ concentration in Yangon City. As a preliminary study, we could not explain exactly the reasons of diurnal variation of PM_2.5_ concentration. The higher PM_2.5_ concentration in the morning and evening in Yangon City suggests traffic-related emission in the rush hours between 7 and 10 am and between 7 and 10 pm. Heavy-duty vehicles emitted PM six times greater than light-duty vehicles [17]. In Yangon, heavy-duty vehicles are allowed access to seven major roads from 9 pm to 6 am, and this traffic restriction may play a role in decreasing PM_2.5_ concentration in afternoon hours. In addition, 12% of total vehicles in Myanmar are used in Yangon [18].

Recent study has indicated that PM_2.5_ concentration measured during 2 months study periods by using CW-HAT 200 handheld particulate matters monitoring device in Mingaladon, one of the crowded townships of Yangon, was 61.48% which was found to be exceeded than WHO reference value [19]. Taken together, it is suggested that the major source of PM_2.5_ in Yangon is traffic vehicle emission. Ambient temperature, humidity, and wind speed may be contributing factors for PM_2.5_-related air pollution in Myanmar.

According to similar study using Pocket PM_2.5_ Sensor in other countries [12], we found a high concentration level (60–83 μg/m^3^) in smoking and grill restaurant zones, which are known as ‘hotspots’ in Tokyo Japan. In Weihai City, China, the PM_2.5_ concentration was around 28–36 μg/m^3^ at the seaside and, in contrast, it was 44–57 μg/m^3^ in downtown. Also, we found a hotspot (94 μg/m^3^) close to an exhaust connected to underground restaurants in Weihai City. In Seoul City, Korea, PM_2.5_ concentration was approximately 47 μg/m^3^ in downtown and 30–35 μg/m^3^ in a garden area. From our findings using Pocket PM_2.5_ Sensor indicate that PM_2.5_ concentration varies with region of sampling areas such as near hotspots, quiet residential areas, or traffic congestion not only in developing country like Myanmar, but also in developed countries like Japan and Korea.

Pocket PM_2.5_ Sensor is a mobile, portable device, easy to carry everywhere, and easy to use with software application. It can be used to measure in several locations and detect in real-time. Ordinal measuring devices are fixed at station and data analysis is required. However, Pocket PM_2.5_ Sensor can be used for 1 h only and ordinal measuring device can be used for 24 h.

Finally, the pocket sensor used in this study is found to be able to record the real-time PM_2.5_ concentration of the ambient air. Pocket PM_2.5_ Sensor has a great potential for mobile citizen sensing and visualization. Its accuracy seems sufficient, but more assurance is needed by performing regular cross-checking with public monitoring instruments. This Pocket PM_2.5_ Sensor would be helpful to use as a personal sampler as well as for evaluation of distribution of PM_2.5_ in local or specific areas in real-time, easily and effectively (Fig. 5).

## Conclusions

The PM_2.5_ level in Yangon City has reached the noticeable level. Within townships, air quality, particularly in relation to concentrations of PM_2.5_, tends to be worse close to busy roads and construction sites. This is the first study to quantify the regional distribution of PM_2.5_. Our future plan is to detect individual exposure screening of PM_2.5_ concentration in highly contaminated area using Pocket PM_2.5_ Sensor and investigate whether association exists between PM_2.5_ concentration and health risk in Myanmar.

## Abbreviations

ANOVA: Analysis of variance
CSV: Comma-separated values
GPS: Global positioning system
KML: Keyhole Markup Language
LED: Light emitting diode
NIES: National Institute for Environmental Studies
PD: Photodiode
PM: Particulate matter
SD: Standard deviation
SPSS: Statistical Package for Social Science
UM 1: University of Medicine 1
